# Nanocellulose aerogels as 3D amyloid templates†

**DOI:** 10.1039/d3nr02109b

**Published:** 2023-11-01

**Authors:** Ashutosh Sinha, Nico Kummer, Tingting Wu, Kevin J. De France, Dorothea Pinotsi, Janine L. Thoma, Peter Fischer, Silvia Campioni, Gustav Nyström

**Affiliations:** a Laboratory for Cellulose and Wood Materials, Empa Überlandstrasse 129 8600 Dübendorf Switzerland Gustav.nystroem@empa.ch Silvia.campioni@empa.ch; b Institute of Food Nutrition and Health, ETH Zürich Schmelzbergstrasse 7 8092 Zürich Switzerland; c Scientific Centre for Optical and Electron Microscopy, ETH Zurich 8093 Zurich Switzerland

## Abstract

Proteins in solution tend to coat solid surfaces upon exposure. Depending on the nature of the surface, the environmental conditions, and the nature of the protein these adsorbed proteins may self-assemble into ordered, fibre-like structures called amyloids. Nanoparticulate surfaces, with their high surface to volume ratio, are particularly favourable to amyloid formation. Most prior research has focussed on either inorganic or organic nanoparticles in solution. In this research, we instead focus on aerogels created from TEMPO-oxidized cellulose nanofibers (TO-CNF) to serve as bio-based, three-dimensional amyloid templates with a tuneable surface chemistry. Previous research on the use of cellulose as a protein adsorption template has shown no evidence of a change in the secondary protein structure. Herein, however, with the aid of the reducing agent TCEP, we were able to induce the formation of amyloid-like ‘worms’ on the surface of TO-CNF aerogels. Furthermore, we demonstrate that the addition of the TO-CNF aerogel can also induce bulk aggregation under conditions where it previously did not exist. Finally, we show that the addition of the aerogel increases the rate of ‘worm’ formation in conditions where previous research has found a long lag-phase. Therefore, TO-CNF aerogels are shown to be excellent templates for inducing ordered protein aggregation.

## Abstract 2

Nanocellulose aerogels induce and accelerate HEWL aggregation, forming β-sheet rich amyloid-like ‘worms’.

## Graphical abstract

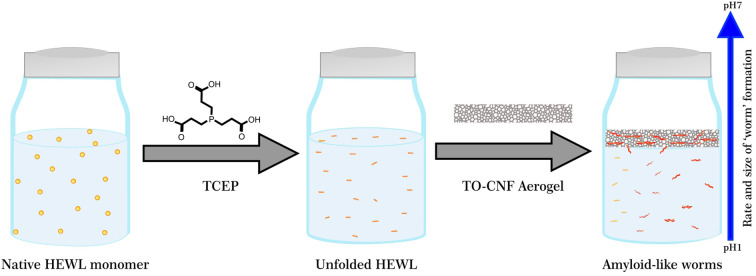

## Introduction

While historically associated solely with diseases such as Alzheimer's and Parkinson's,^1^ amyloid proteins also perform useful and vital functions in various organisms.^2^ Fungal hydrophobins, for example, allow the organism to attach to host substrates, form aerial structures, and protect fungal spores from the host immune system.^3,4^ Over the past two decades, amyloids from non-pathogenic proteins have also been used to create a myriad of materials with functions ranging from heavy metal capture to underwater adhesives to substrates for cell culture.^5–7^ Irrespective of the pathology or utility of amyloids, their mechanism of formation and the factors influencing it have piqued research interest for decades.

One such factor that has recently gained traction is the role of surfaces, particularly nanoparticle surfaces. Upon exposure to certain surfaces, the protein adsorbs onto them forming a coating and a so-called nanoparticle-protein ‘corona’.^8^ This may cause an acceleration or inhibition of protein aggregation.^9^ The precise nature of this interaction depends on factors intrinsic to the protein, the structure and composition of the surface, as well as the environmental conditions during which the interaction occurs (pH, temperature, salt concentration, *etc*.). Both, the amino acid sequence of the protein and the intrinsic protein stability often determine whether the presence of nanoparticles will accelerate or inhibit its aggregation. For example, amyloid formation in protein mutants with high intrinsic stability and low propensity to aggregate was accelerated by the presence of nanoparticles.^10^ Amyloid formation in mutants with low stability and high aggregation propensity on the other hand was inhibited by their presence.^10^

The most crucial factor, however, is the nature of the surface introduced.^11^ A rough surface, for instance, limits diffusion of the Amyloid-β peptide and thereby limits its self-assembly into fibrils.^12^ Ideally, a surface attracts protein monomers onto itself and the resultant decrease in the surface energy required for nucleus formation, coupled with the increase in monomer concentration accelerates the fibrillation process.^13^ However, if surface attraction is too strong, the mobility of these monomers is inhibited and so is the formation of stable fibrils for proteins with a high bulk aggregation propensity.^14^ The nucleation rate, therefore can be understood as a product of both, monomer concentration and aggregation propensity. A surface with a higher nucleation rate than the bulk shall therefore accelerate aggregation, while a surface with a lower nucleation rate than the bulk shall inhibit it.^15^

Nanoparticle surfaces introduce additional variables to the equation. Smaller spherical nanoparticles (5 nm diameter), with resultant high surface curvature inhibit amyloid formation by destabilizing peptide oligomers in contrast to larger nanoparticles (20 nm).^16^ Research on the role of nanoparticle surfaces in amyloid formation has found varied results, ranging from inhibition, to acceleration, or indifference.^17–19^ This research has focused mainly on the role of spherical inorganic/organic nanoparticles dispersed in solution. However, three-dimensional objects created from nanoparticles could also serve as an excellent template for amyloid formation. The resultant biohybrid structure could possess the mechanical strength offered by the 3D template and the functionality offered by the protein. The ideal solid template should offer the highest possible surface area, yet limit surface curvature.^16^ Preferably, we should be able to tune its surface chemistry and charge according to our needs, allowing us to explore ideal conditions for amyloid formation. Additionally, if the nanoparticulate material was bio-based, biocompatible, and biodegradable, the horizons for such a biohybrid material would be much broader. We accomplished this by turning to the most abundant biopolymer on Earth, cellulose.

Through mechanical and chemical processes, cellulose can be converted to cellulose nanofibers (CNFs) with a diameter of around 20 nm and length in the micrometre range.^20^ CNFs are high aspect ratio particles that can be physically or chemically cross-linked to yield three-dimensional gel networks, that can be further processed into ultra-lightweight and porous structures known as aerogels.^21,22^ The process of TEMPO-mediated oxidation can introduce carboxyl groups on the surface of the CNFs and thus, in turn, the aerogel (TO-CNF aerogels).^23^ The resultant negative charge can be controlled by the degree of oxidation, and be utilized to electrostatically attract positively charged proteins, immobilizing them on the aerogel surface. To study the protein interaction with the TO-CNF aerogels, we selected the functional protein hen egg white lysozyme (HEWL). HEWL remains positively charged in solution across a wide range of pH values, owing to its high isoelectric point (∼11).^24^ Previously, our group has created biohybrid aerogels consisting of HEWL amyloids and TO-CNF.^25^ Monomeric HEWL (native as well as denatured), HEWL-derived peptides, and aggregates of HEWL (amyloid fibrils as well as globular oligomers with amyloid-like properties) display antimicrobial activity against bacteria and fungi.^26–30^ Notably, HEWL monomers that were immobilized on TO-CNF aerogels saw a marked reduction in their antimicrobial activity.^31^ The study theorized that the reduction in activity was due to the interaction between the positively charged lysozyme and the negatively charged aerogel, hindering its active sites and disrupting enzyme freedom. This strong electrostatic attraction could also induce unfolding of the protein. Research on regenerated cellulose surfaces (without TO-oxidation) that analysed the secondary structure of HEWL monomers adsorbed found no change from the monomeric to aggregated forms.^32^ At other interfaces, such as the oil–water interface, the adsorption of the lysozyme did not seem to result in much denaturation.^33^ However, prior exposure to a reducing agent, DTT, resulted in stable interfacial films, possibly indicating aggregation.

In this research, we aim to determine the ability of TO-CNF aerogels to act as templates for HEWL amyloid formation. The electrostatic attraction between the oppositely charged aerogel and protein as well as its large surface area, should provide ideal conditions for amyloid formation. Additionally, we evaluated the potential of catalysts such as the reducing agent TCEP, which causes unfolding of the HEWL monomer, potentially accelerating amyloid formation.^26^

## Materials and methods

### Preparation of TO-CNF aerogels

TO-CNF aerogels were prepared as previously described.^22^ Briefly, TEMPO and NaBr solutions of 0.1 and 1.0 mmol per gram of cellulose pulp respectively, were added to a 2% dispersion of never-dried cellulose pulp. The pH was initially maintained between 10 and 10.5. This was followed by the addition of 10 mmol of sodium hypochlorite per gram of cellulose. The oxidized fibres were then washed several times, ground using a Supermass Colloider, and further fibrillated using a high-shear homogenization process. The desired concentrations of CNF fibril suspensions were obtained *via* rotary evaporation. To form the aerogels, 1.5 wt% of TEMPO-CNF suspension in a Petri dish was then placed in a container with a few drops of 37% HCl. This container was placed in an oven at 70 °C for 3 h to create a saturated HCl atmosphere, which protonated the carboxyl groups of the TEMPO-CNF and led to physical cross-links. This was followed by freeze casting in liquid nitrogen, thawing cycles in ethanol, and ultimately supercritical drying to yield the final dual-porosity aerogels.^22^

### Aerogels as 3D amyloid templates

2 mg mL^−1^ HEWL (lysozyme from chicken egg white, Sigma Aldrich) solutions in MilliQ water were mixed in a 1 : 1 volume ratio with either 5 mL MilliQ or 5 mL of 25 mM TCEP. Solutions containing TCEP were adjusted to final pH values of 2.5, 3.5 and 4.5 using 0.1 M NaOH. Aerogels were then cut into 10 mg pieces and incubated at room temperature in vials with the prepared HEWL-TCEP solutions for 24 hours. Aerogels were then removed and dried overnight in ambient conditions (for FTIR analysis and SEM) or transferred to a staining solution (for TIRF microscopy). FTIR analysis was performed using a Bruker Tensor 27 FT-IR in attenuated total reflectance (ATR) mode. All spectra were recorded between 4000 and 600 cm^−1^ at a resolution of 4 cm^−1^ with 32 scans per sample, followed by a background correction. A TO-CNF suspension mixed with HEWL in a 1 : 1 ratio along with 12.5 mM TCEP was also incubated as a control. The spectra were normalised to the C–O peak of cellulose to account for the variance in the area of the aerogel exposed to the IR beam. The spectra were then deconvolved with the aid of the Fourier self-deconvolution tool in OriginLab® at a gamma value of 60 and a smoothing factor of 0.2. The quantification of secondary structures was performed as previously established by using a Gaussian curve fitting process, since the samples were solids.^34,35^

### Detecting bulk aggregation

HEWL solutions incubated with aerogels were observed for 24 hours. Any visible turbidity that developed in the solutions over time was considered an indication of micrometre scale protein aggregation. Solutions that remained visually clear were evaluated by DLS to detect aggregation at the nanometre scale. Similar to Kummer *et al*. 2021, A Malvern Zetasizer Nano ZS was used for time-resolved DLS to evaluate the aggregation kinetics.^26^ The prepared TCEP-HEWL solutions were filtered through a 0.02 μm Whatman syringe filter into a cuvette. A piece of the TO-CNF aerogel was rinsed in MilliQ to get rid of any loosely attached CNF fibres and placed on the solution surface in the cuvette. The *Z*-average diameter in solution was measured at an interval of 15 minutes for 24 hours. The clear solutions were also imaged *via* AFM to visualize these protein aggregates (if any).

### Microscopic investigations

Atomic force microscopy in tapping mode was performed on solutions after completion of DLS measurements on a Bruker Icon 3 equipped with a Bruker RTESPA-150 probe. The samples were deposited on a freshly cleaved mica, rinsed with MilliQ, dried using pressurized air, and imaged at a scan rate of 0.5 Hz and a resolution of 1024 lines. Aerogels incubated with pH 2.5 HEWL-TCEP solutions were selected for TIRF microscopy and SEM imaging. For TIRF microscopy, aerogels were removed 24 hours post incubation and transferred to a solution containing 10 μM FSB dye in MilliQ for 3 hours. The standard amyloid dye, Thioflavin-T, was not selected as it stained cellulose fibres as well. The aerogels were then washed thrice in MilliQ for 15 minutes each and imaged in TIRF or Highly Inclined (HILO) illumination mode, using a Nikon N-STORM microscope (Nikon, UK Ltd) and an SR Apochromat TIRF 100× 1.49 NA oil immersion objective lens. Fluorescence was detected with an EM-CCD Camera iXon DU897 (Andor). The field of view imaged typically covered 512 × 512 camera pixels corresponding to an area on the sample of ∼80 × 80 μm^2^. An in-built focus-lock system was used to prevent axial drift of the sample during data acquisition. The laser excitation was at 20% or 30% at 405 nm, of a maximum intensity measured at the tip of the optical fibre of 20 mW and with an exposure time of 50 milliseconds. The emission passed through a multi-edge dichroic filter with windows at 415–490 nm, 502–560 nm, and 660–800 nm. Images were acquired using the NIS Elements Nikon software. For SEM, aerogels were removed after 24 hours of incubation at pH 2.5 and dried overnight. They were then coated with a 20 nm thick layer of carbon, and imaged in a FEI Quanta 650 FEG ESEM microscope.

## Results and discussion

### Aerogels accelerate bulk aggregation

**Fig. 1:**
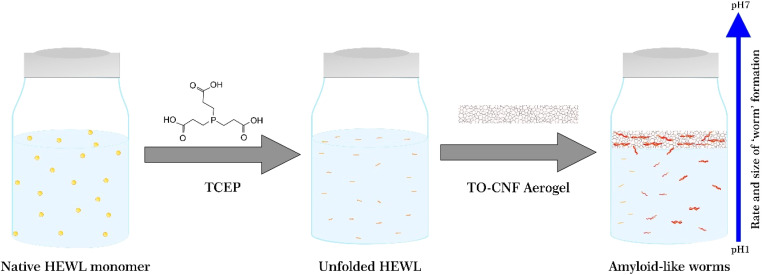
Schematic illustrating the unfolding, aggregation, and self-assembly of the HEWL monomer in the presence of TCEP and the TO-CNF aerogel and as a function of varying pH.

To observe the impact of TO-CNF aerogels on the bulk aggregation of HEWL, aerogels were incubated in HEWL-TCEP solutions containing 1 mg mL^−1^ HEWL, 12.5 mM TCEP as schematically shown in Fig. 1. The pH of the samples was adjusted to 2.5, 3.5, or 4.5. Previous research suggests that at pH values of 5.5 and above, TCEP-induced aggregation results in large, three-dimensional, micrometre-scale particles producing visible turbidity in the HEWL solution.^36^ At a lower pH value of 4.5, aggregation is on the other hand limited to one dimension, in the nanometre scale, has a long lag phase, no visible turbidity, and results in the formation of amyloid-like ‘worms’.^26^

**Fig. 2:**
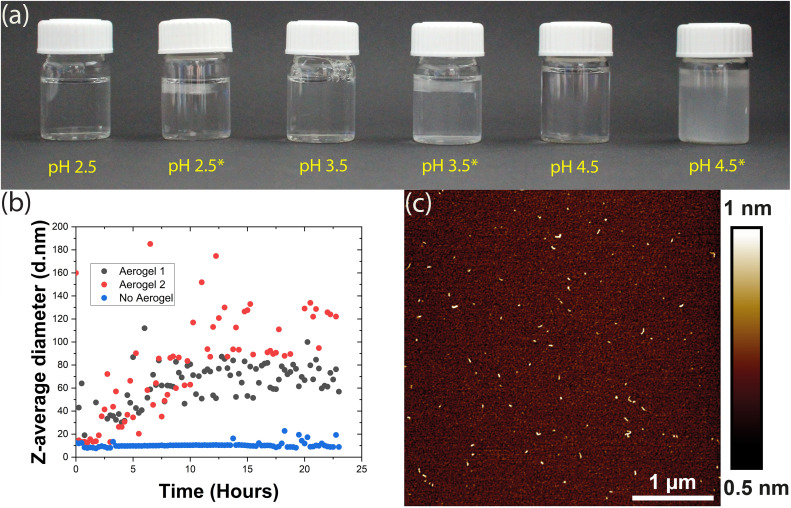
(a) HEWL-TCEP solutions at various pH values in the presence and absence of the TO-CNF aerogel. Solutions with the aerogel are marked with an asterisk (*). (b) *Z*-Average diameter of the particles dispersed in the HEWL-TCEP solution at pH 3.5 incubated in the presence (black and red) and absence (blue) of the aerogel. Black and red data correspond to two independent replicates. (c) AFM height image of the aggregates found in the HEWL-TCEP solution at pH 3.5 incubated for 24 hours in the presence of the aerogel.

However, when we incubated 1 mg mL^−1^ HEWL and 12.5 mM TCEP with a TEMPO-CNF aerogel at pH 4.5, the sample began to turn turbid almost instantly. Tiny light scattering particles were initially observed as a band near the surface of the aerogel and then, within an hour, throughout the vial. These particles agglomerated over time to form larger particles that initially remained dispersed in the vial (Fig. 2a), but eventually sedimented. This gave a first clear indication that the aerogel significantly accelerated the aggregation process, producing larger light scattering particles. Encouraged by these results, we therefore investigated this phenomenon at even lower pH values. Here, as the solutions did not display visible changes in turbidity, aggregation was monitored *via* dynamic light scattering (DLS). Briefly, HEWL-TCEP solutions were filtered into a cuvette and a small piece of the TEMPO-CNF aerogel was placed on its top. The *Z*-average diameter of the particles present in solution was then measured overnight.

At pH 2.5, the presence or absence of the aerogel did not affect bulk aggregation. In both cases, DLS did not detect any significant changes in the *Z*-average diameter of the particles in the HEWL solution (ESI Fig. 1†). Occasionally, a larger particle was observed as an outlier. At pH 3.5, no significant bulk aggregation was detected by DLS in the absence of the aerogel (Fig. 2b). However, when exposed to aerogel templates, HEWL began to aggregate in the solution, albeit at the nanometre scale. Occasionally, DLS measurements indicated large *Z*-average diameters in the micrometre scale (ESI Fig. 2†). This was attributed to larger worm-like fibres that form on the surface of the aerogel and then detach. If these outliers were removed, a steady increase in the size of bulk aggregates could be observed, possibly reaching a plateau 16 hours into the experiment (Fig. 2b). These aggregates were visualized by AFM and appeared to be similar to the amyloid-like worms described above (Fig. 2c). Thus, the addition of the aerogel resulted in the formation of amyloid-like worms in the bulk, a few nanometres in size.

Overall, the presence of the TEMPO-CNF aerogel apparently lowers the pH value at which HEWL aggregation into amyloid-like particles can occur. Our observations can be rationalized by taking into account the surface charge of the aerogel. The carboxyl groups present on the aerogel surface are completely deprotonated at pH values higher than their p*K*_a_ (∼4), making the aerogel highly negatively charged. At around pH 3.5, roughly 50% of the carboxyl group may be deprotonated resulting in a moderate negative charge, whereas at pH 2.5 the aerogel may at best possess a mild negative charge.^37^ The resultant surfaces would thus range from weakly attractive (pH 2.5) to strongly attractive (pH 4.5) for the HEWL species. With increased monomer concentration at the moderately attractive surface, the rate of nucleation increases, leading to an acceleration of amyloid-like worm formation.^13^ The aggregates formed on the surface of the aerogel could then detach and become dispersed in bulk. However, this would mean that these aggregates detached spontaneously from an oppositely charged surface, since samples were not mechanically agitated. If this argument is to be believed and gravitational forces are the primary reason for such detachment, this phenomenon should be size dependent.

At pH 4.5 where nucleation and worm formation occurred even in the absence of the aerogel, the highly charged surface further accelerates this process and causes large aggregates to form. These aggregates were first visually observed as a band near the aerogel, indicating spontaneous detachment from its surface. Therefore, the more attractive surfaces at pH values of 3.5 and 4.5 might form larger and more worms on the aerogel surfaces, leading to a higher rate of detachment. The detached amyloid-like particles may then act as a nucleation site for further aggregation. This could explain the DLS measurements that show a gradual increase in *Z*-average diameter over time observed in Fig. 2b. Previous research has also demonstrated the tendency of TO-CNF to ‘split’ resulting in nanoscale segments detaching from the larger fibril.^38^ This may in turn result in the heterogeneous nucleation of the HEWL monomer, offering an alternative explanation.^39^ At a lower pH (2.5), the low charge on the aerogel surface is not enough to rapidly accelerate aggregation, nor is the aggregation propensity in the bulk particularly high.^13^

### Aerogels induce aggregation and amyloid formation on their surface

**Fig. 3:**
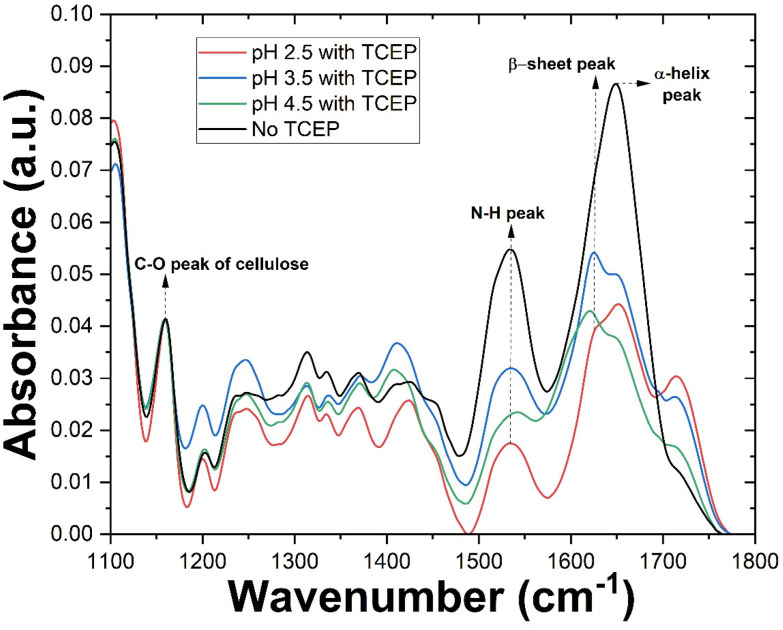
IR spectra of various TO-CNF aerogel incubated in HEWL solutions with or without TCEP at various pH values. The spectra were normalized to the C–O peak of cellulose. Arrows indicate the positions of different peaks and the corresponding vibration.

Following investigations of TO-CNF aerogel-catalysed bulk aggregation, we turned our attention to characterizing this phenomenon at the aerogel surface. TO-CNF aerogels suspended in HEWL-TCEP solutions at various pH values for 24 hours were removed, dried overnight, and assessed *via* ATR-FTIR. Control samples prepared without TCEP at pH values of 2.5 and 4.5 were also measured (ESI Fig. 3†). As suspected, upon exposure to the oppositely charged aerogel, the monomeric lysozyme is immobilized on its surface and yields a large peak at ∼1640 cm^−1^ (Fig. 3). This peak is indicative of the presence of α-helix structures and is similar to the peak found in native HEWL structures alone (ESI Fig. 3†). However, our preliminary observations noted little evidence of amyloid-like aggregates, *i.e.* which should be evident from the appearance of β-sheets. Previous research has demonstrated that an FTIR investigation of proteins rich in cross β-sheets displays a peak around 1625 cm^−1^.^35^ After incubation for just 24 hours, no such peak was initially observed in absence of TCEP. The possibility of an even longer incubation time resulting in amyloid formation on the surface of the aerogel even in absence of TCEP, however, cannot be completely ruled out.

As discussed in the previous section, at pH 2.5 no bulk aggregation was observed in our experiments. In the FTIR spectrum of the aerogel surface however, in addition to the α-helix peak, the spectrum also displayed a prominent peak at ∼1625 cm^−1^, indicative of β-sheets. The larger peak at ∼1640 cm^−1^ indicates that while disordered aggregation and residual monomers account for a greater share of the protein after 24 hours, a significant portion is present as β-sheeted aggregates. Thus at pH 2.5, aggregation takes place only on the aerogel surface. A similar result was observed when a dilute TO-CNF suspension was incubated with HEWL/TCEP (ESI Fig. 3†).

Additionally, we aimed to quantify various secondary structures in these conditions. Following previously established procedures,^34,35^ we deconvolved each peak (ESI Fig. 4†). Savitzky–Golay smoothed second derivative curves of each sample (ESI Fig. 5†) were then plotted and peaks of the deconvolved spectra were used as references. Finally, the area under the peak of each Gaussian-fitted second derivative curve was used to approximate secondary structure content. The sample in the absence of TCEP showed similar levels of α-helix and β-sheet structures at approximately 38 and 36% respectively. Also, a significant portion of the secondary structures consisted of β-coils at approximately 24%. However, the β-sheet content in this case may be overestimated due to the large width of the curve (ESI Fig. 5a†) causing significant overlap with the region of random secondary structures. Upon the addition of TCEP, however this changes drastically (ESI Fig. 5b†). A sharp peak in the β-sheet region of the curve resulted in β-sheets accounting for nearly 46% of secondary structures. α-Helical structures accounted for a similar 48% of secondary structures. This can be explained by the destabilisation of the protein structure due the cleavage of disulphide bonds caused by TCEP.^26^ At higher pH values in the presence of TCEP, β-sheets became the dominant secondary structure. However, there appears to be an upper limit for β-sheet content in this case, with samples at pH 3.5 and 4.5 reporting similar results at 58 and 55% respectively (ESI Fig. 5c and d†).

At both pH 3.5 and pH 4.5, amyloid formation occurred in the bulk as well as on the aerogel surface. Therefore, distinguishing amyloids formed at the surface from those formed in the bulk and potentially later adsorbed onto the aerogel surface would be a difficult task. To investigate the templating effect of the aerogels, we therefore selected the condition that resulted in amyloid formation only on the surface of the aerogel (pH 2.5). As controls, aerogels were also incubated in solutions containing no TCEP and no HEWL.

### Visualizing amyloids on the aerogel surface

**Fig. 4:**
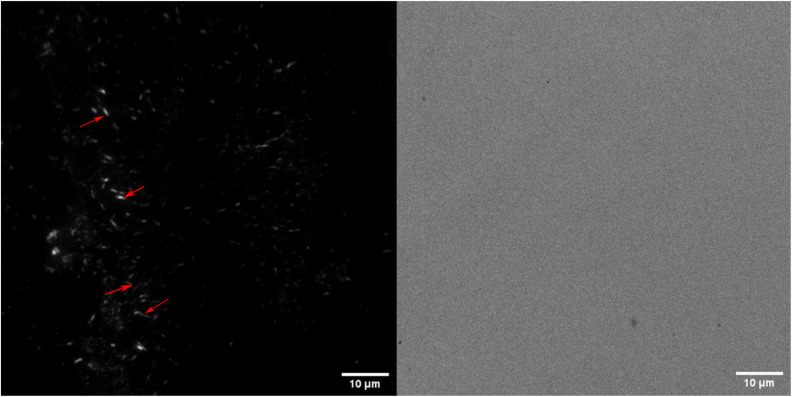
TIRF (left) and Brightfield (right) images of TO-CNF aerogels incubated in HEWL-TCEP solutions at pH 2.5 stained with FSB. Red arrows indicate various worm-like protein fibres formed on the aerogel surface.

Aerogels were incubated in their respective solutions for 24 hours and then transferred to a solution containing 10 μM FSB stain in MilliQ for 3 hours. The samples were then washed thrice for 15 minutes in MilliQ with gentle mixing to remove any free stain and evaluated *via* TIRF microscopy. The surface of TO-CNF aerogels incubated in HEWL-TCEP solutions contained various, nanoscale, wormlike fibres (Fig. 4). The fibres reached a length of a couple of microns and emitted a strong fluorescence signal. These more fibre-like aggregates could clearly be distinguished from more amorphous structures observed in conditions where no TCEP was present (ESI Fig. 6†). We also observed some fluorescence emitted from aerogels incubated in the absence of protein. However, these structures were not fibre-like or were also visible in the Brightfield (ESI Fig. 6†). Therefore, they were deemed to be cracks or artefacts on the aerogel surface that trapped the stain rather than the result of its amyloid-specific dye binding.

**Fig. 5:**
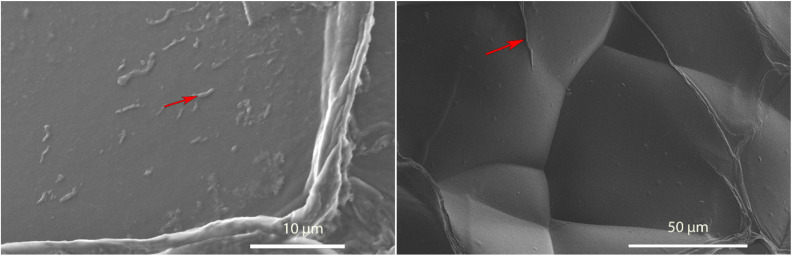
SEM image of a TO-CNF aerogel incubated in a HEWL-TCEP (left) or TCEP solution (right) at pH 2.5 for 24 hours. Red arrows indicate a fibre-like structure on the surface of each aerogel that was investigated *via* EDX spectroscopy.

We confirmed this *via* SEM examinations of aerogels incubated under the same conditions. SEM revealed worm-like structures attached to the surface of the aerogel with lengths of a few micrometres (Fig. 5). Worm-like aggregates could not be seen on the surface of aerogel samples incubated without either HEWL or TCEP (ESI Fig. 7†). Elemental analysis *via* EDX spectroscopy also revealed a nitrogen content of between 13–16%, which is equivalent to the nitrogen content in proteins (Table 1).^40^ In the control without HEWL, no nitrogen was detected, indicating an absence of the protein. Therefore, we can conclude that TO-CNF aerogels acted as 3D templates for the formation of these amyloid fibre-like worms.

**Table 1:** EDX spectroscopic analysis of fibre-like structures on each aerogel

Sample	Carbon content (%)	Nitrogen content (%)	Oxygen content (%)	Trace element content (%)
Aerogel, TCEP	58.49	Not detected	41.51	Not detected
Aerogel, HEWL, TCEP	55.04	15.84	25.49	3.63

## Conclusion

Whether surfaces catalyse or inhibit protein aggregation is specific to individual combinations of proteins, surfaces, and environmental conditions. We evaluated the ability of TEMPO-oxidized CNF aerogels to act as three-dimensional solid templates for the formation of HEWL amyloids. Previous research had indicated that adsorption on cellulose surfaces does not induce changes in the secondary structure of proteins.^32^ While our investigations seem to confirm these results, we attempted to bring about this change with the addition of the reducing agent TCEP. After TCEP destabilized its globular fold, the HEWL monomer was electrostatically attracted to the aerogel surface where the large surface-to-volume ratio provided the ideal conditions for the proliferation of amyloid-like fibrils. At pH 2.5, this was purely a surface phenomenon and amyloid-like fibres were observed on the aerogel surface, but not in bulk. At pH 3.5 and 4.5 instead, amyloid fibres were found on the aerogel surface as well as in bulk at the nanometre and micrometre scale, respectively. While at first glance this was considered the result of amyloids detaching from the aerogel surface, DLS measurements at pH 3.5 showed a gradual increase in protein aggregate size over time, suggesting that aggregation-competent species detached from the aerogel keep attracting monomers in bulk. In the absence of the aerogel, no significant bulk aggregation was observed at pH 2.5 or 3.5. Therefore, TO-CNF aerogels were found to not only act as templates for amyloid formation, but also to accelerate bulk aggregation.

## Author contributions

G.N. and S.C. conceived the experiments and along with P.F. acquired funding for the research. A.S, N.K, K.J.D., D.P and T.W. carried out the experiments. A.S. and J.T. analysed the data, and A.S. wrote the manuscript with input from all the authors.

## Abbreviations

TO: 2,2,6,6-Tetramethylpiperidinyloxy
CNF: Cellulose nanofibers
HEWL: Hen egg white lysozyme
SPG: Schizophyllan
AFM: Atomic force microscopy
DLS: Dynamic light scattering
TCEP: Tris(2-carboxyethyl)phosphine
ThT: Thioflavin T
FTIR: Fourier Transform Infra-red
DTT: Dithiotreitol

## Conflicts of interest

The authors declare no competing financial interests.

## Supplementary Material

NR-015-D3NR02109B-s001
