# Supplementary material for: Nanocellulose aerogels as 3D amyloid templates
Source: Nanoscale. 2023 Nov 1;15(44):17785–92. doi: 10.1039/d3nr02109b (PMC10653027; doi:10.1039/d3nr02109b)
Supplement: NR-015-D3NR02109B-s001 [file NR-015-D3NR02109B-s001.pdf]

Electronic Supplementary Material (ESI) for Nanoscale.
This journal is © The Royal Society of Chemistry 2023

Nanocellulose aerogels as 3D amyloid templates

Ashutosh Sinha [ab], Nico Kummer [ab], Tingting Wu [a], Kevin J. De France [a], Dorothea Pinotsi [c], Peter Fischer [b], Silvia Campioni* [a], Gustav Nyström* [ab]

a. Laboratory for Cellulose and Wood Materials, Empa, Überlandstrasse 129, 8600 Dübendorf.
b. Institute of Food Nutrition and Health, ETH Zürich, Schmelzbergstrasse 7, 8092 Zürich.
c. Scientific Center for Optical and Electron Microscopy, ETH Zurich, 8093, Zurich, Switzerland

1. DLS at pH 2.5

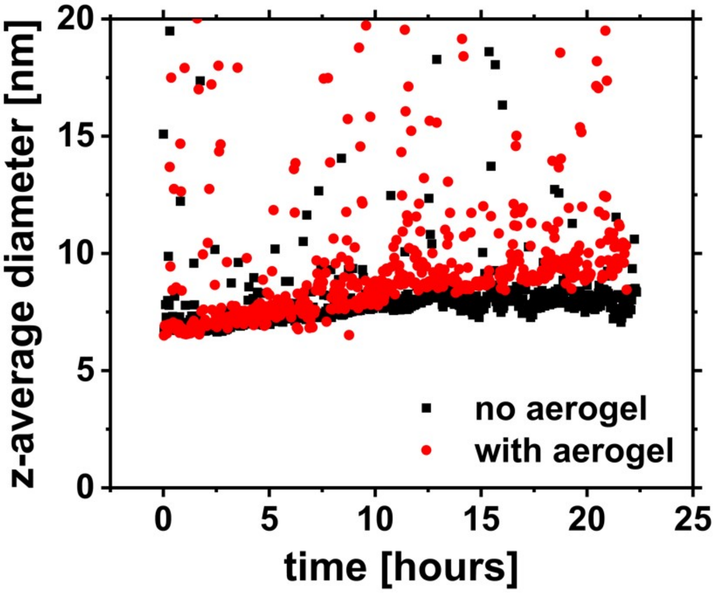

Figure 1: DLS of HEWL-TCEP solution at pH 2.5 with and without TO-CNF aerogel

2. DLS at pH 3.5

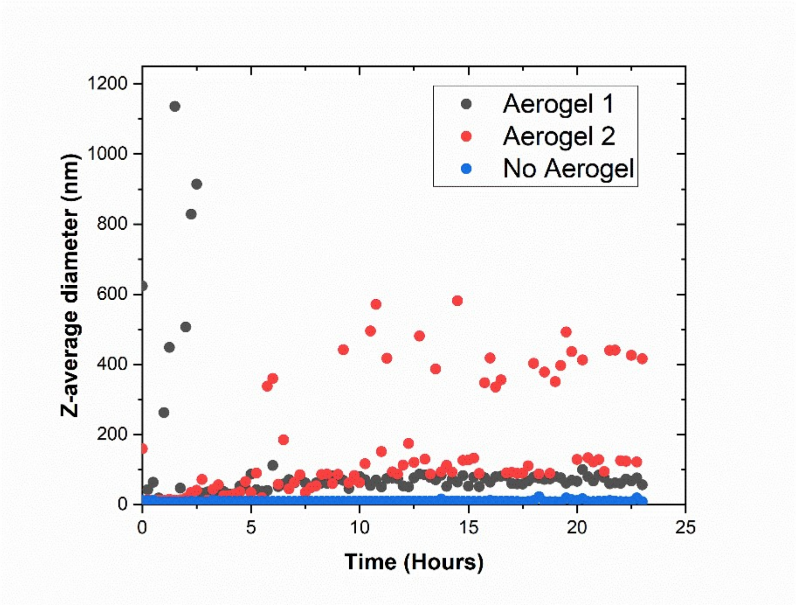

Figure 2: DLS of HEWL-TCEP solution at pH 3.5 with and without TO-CNF aerogel

3. ATR-FTIR analysis of TO-CNF aerogels incubated in HEWL solutions in various conditions

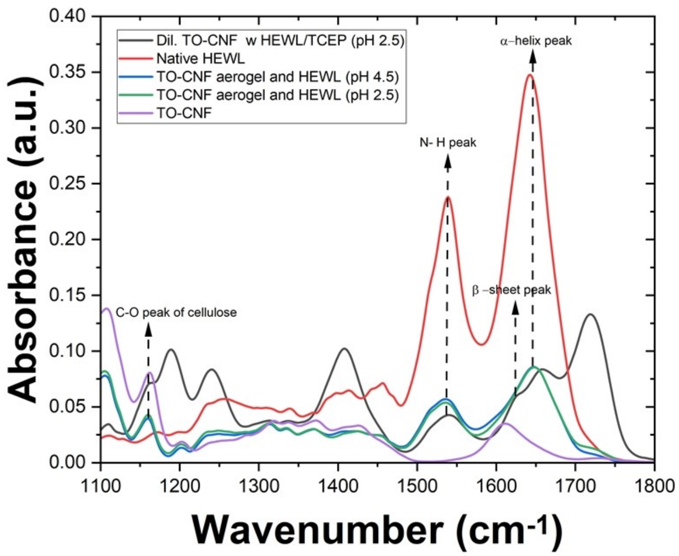

Figure 3: IR spectra of samples incubated under various conditions. Arrows indicate the positions of different peaks and the corresponding vibration.

4. Deconvolution peaks of each FTIR spectra

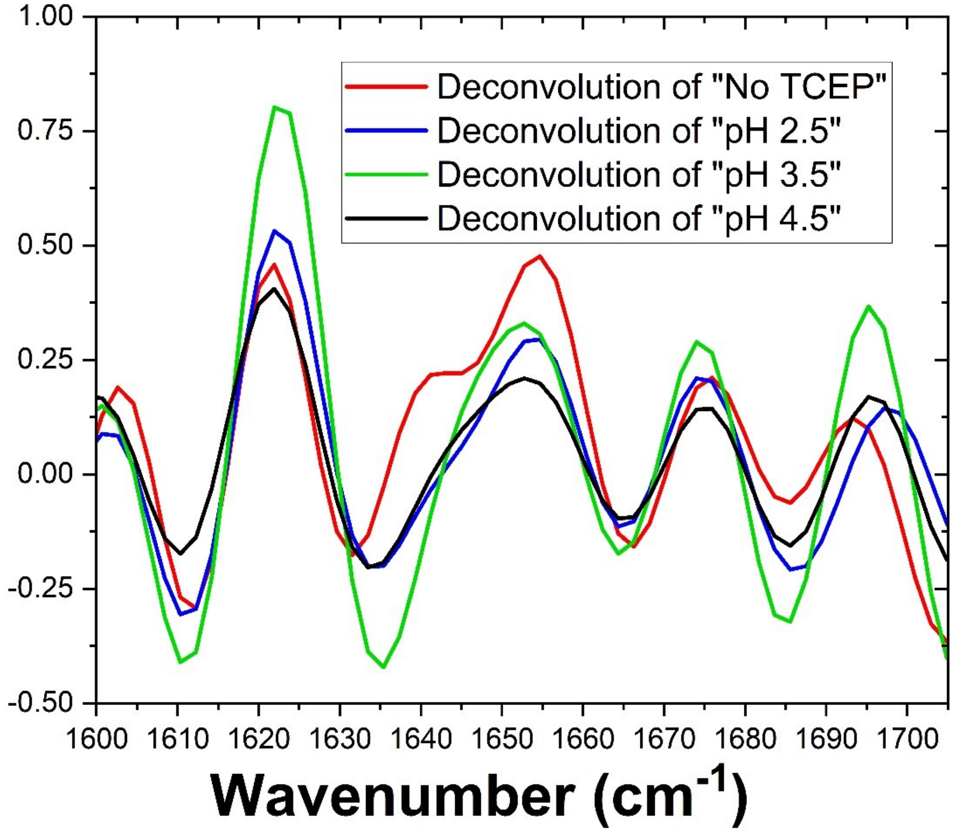

Figure 4: Deconvolution peaks of each FTIR spectra, without TCEP (red curve) and with TCEP and at different pH (blue, green, black curves).

5. Gaussian curve fits of second derivative of various spectra

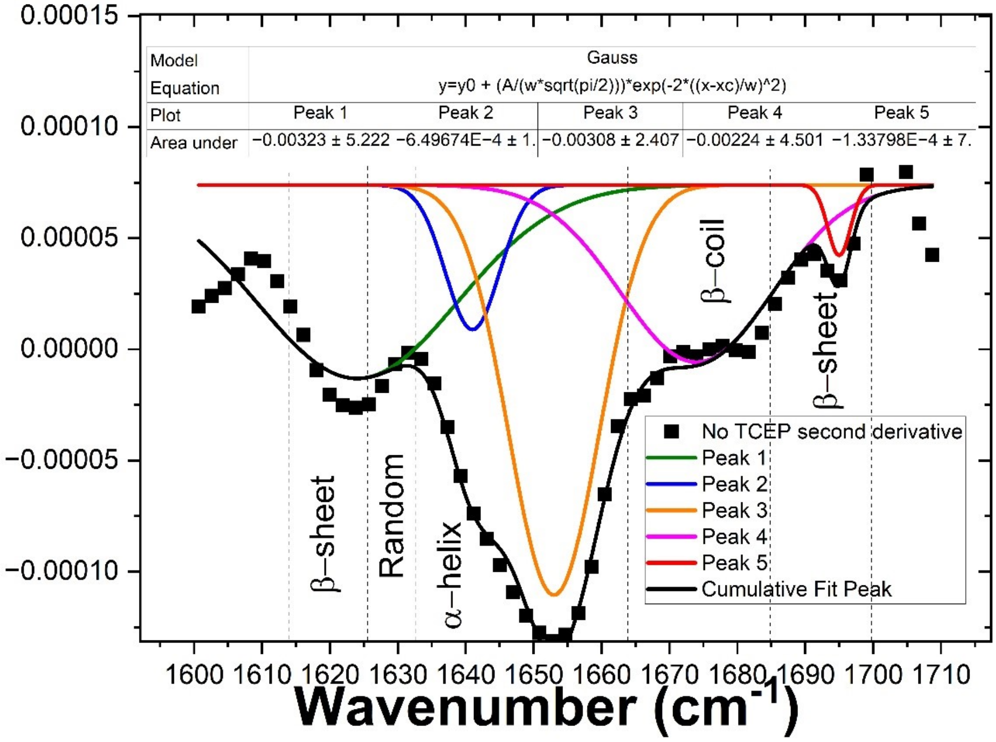

Figure 5a: Gaussian fits of the second derivative of no TCEP spectra.

β-sheet Content% = [Σ(Area of β-sheet curves)/Σ(Area under all curves)] x 100

= 36.01%

Similarly,

α-helix Content% = 39.97%

β-coil Content% = 24.01%

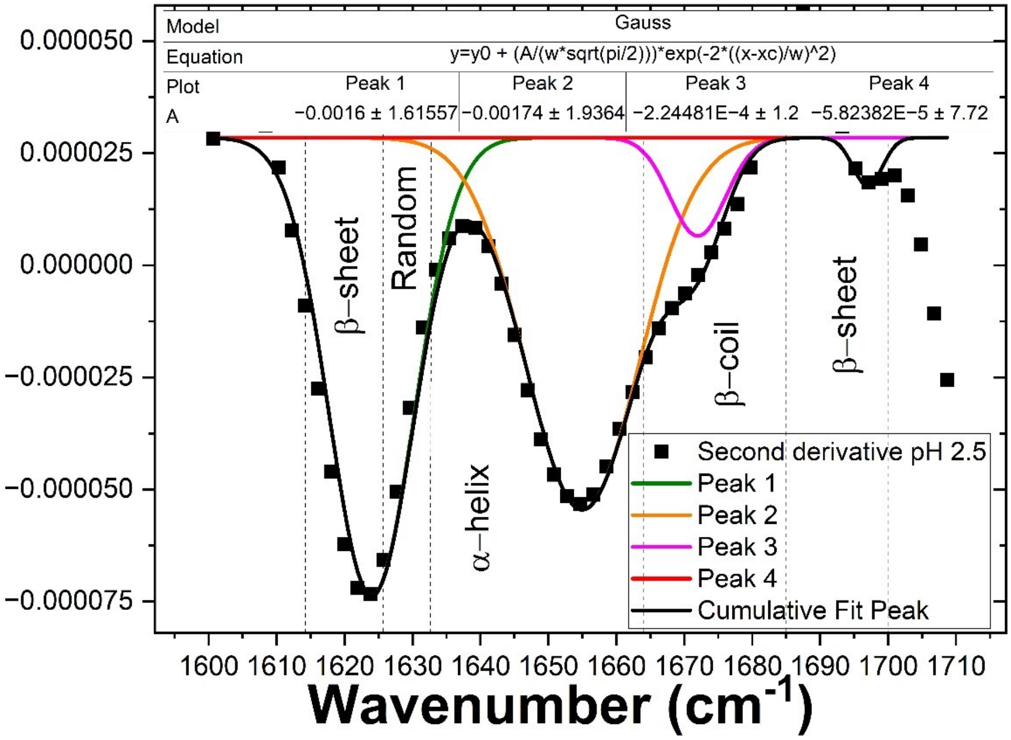

Figure 5b: Gaussian fits of the second derivative of TCEP pH 2.5 spectra.

β-sheet Content% = [Σ(Area of β-sheet curves)/Σ(Area under all curves)] x 100

= 45.86%

Similarly,

α-helix Content% = 48.07%

β-coil Content% = 6.08%

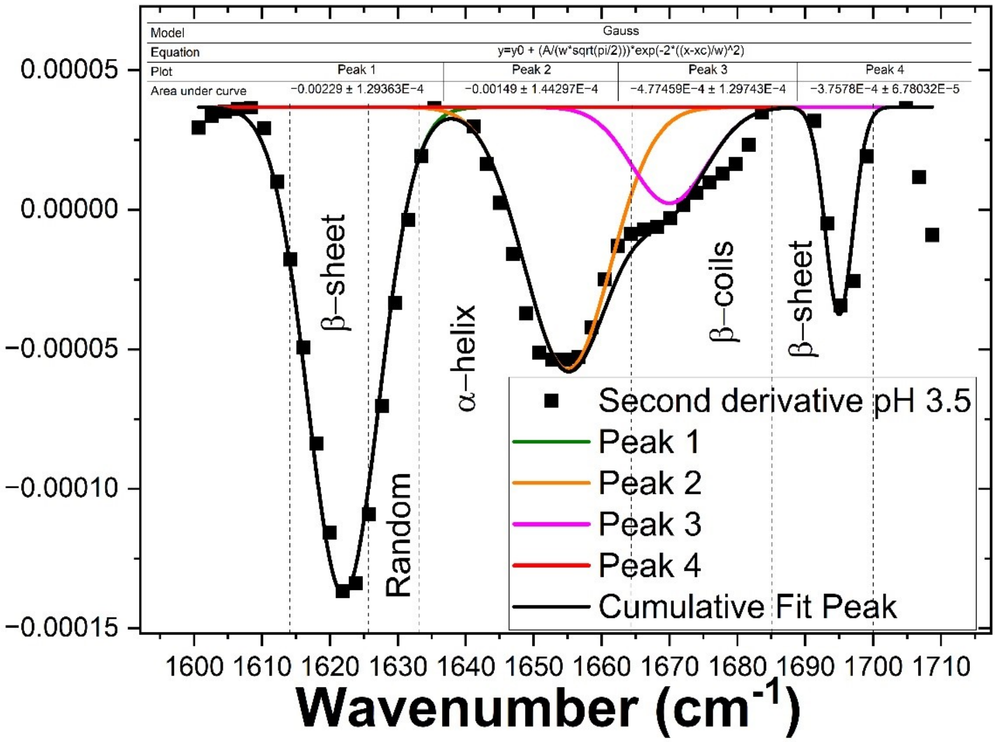

Figure 5c: Gaussian fits of the second derivative of TCEP pH 3.5 spectra.

β-sheet Content% = [Σ(Area of β-sheet curves)/Σ(Area under all curves)] x 100

= 57.79%

Similarly,

α-helix Content% = 32.25%

β-coil Content% = 10.39%

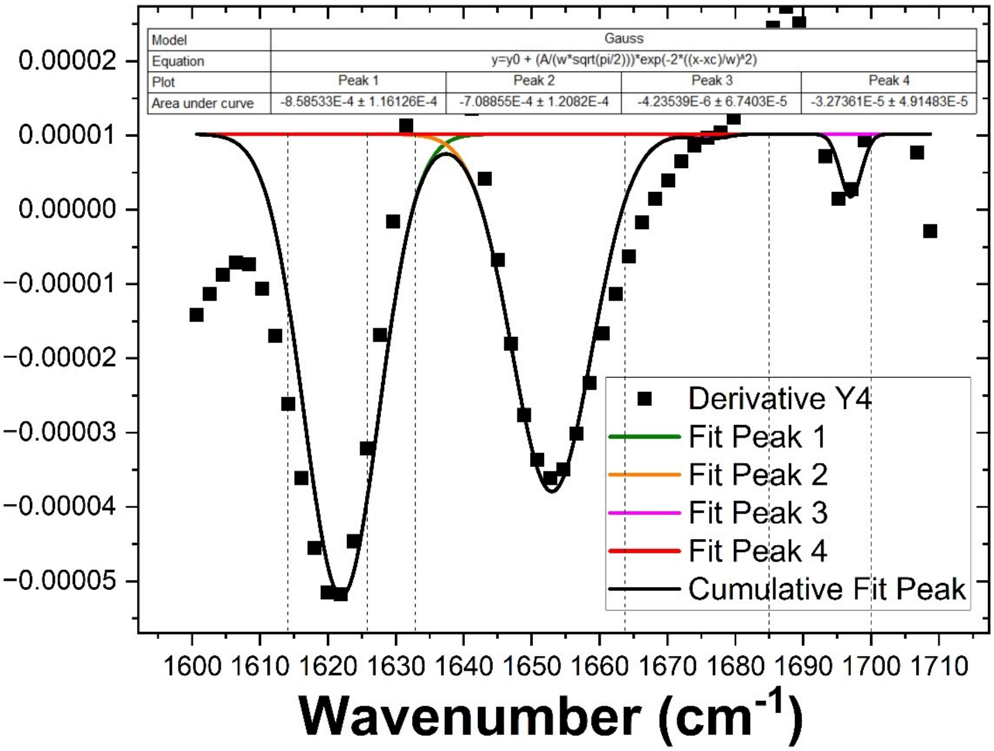

Figure 5d: Gaussian fits of the second derivative of TCEP pH 4.5 spectra.

β-sheet Content% = [Σ(Area of β-sheet curves)/Σ(Area under all curves)] x 100

= 55.56%

Similarly,

α-helix Content% = 44.23%

β-coil Content% = 2.62%

6. TIRF Microscopic investigations on aerogel samples

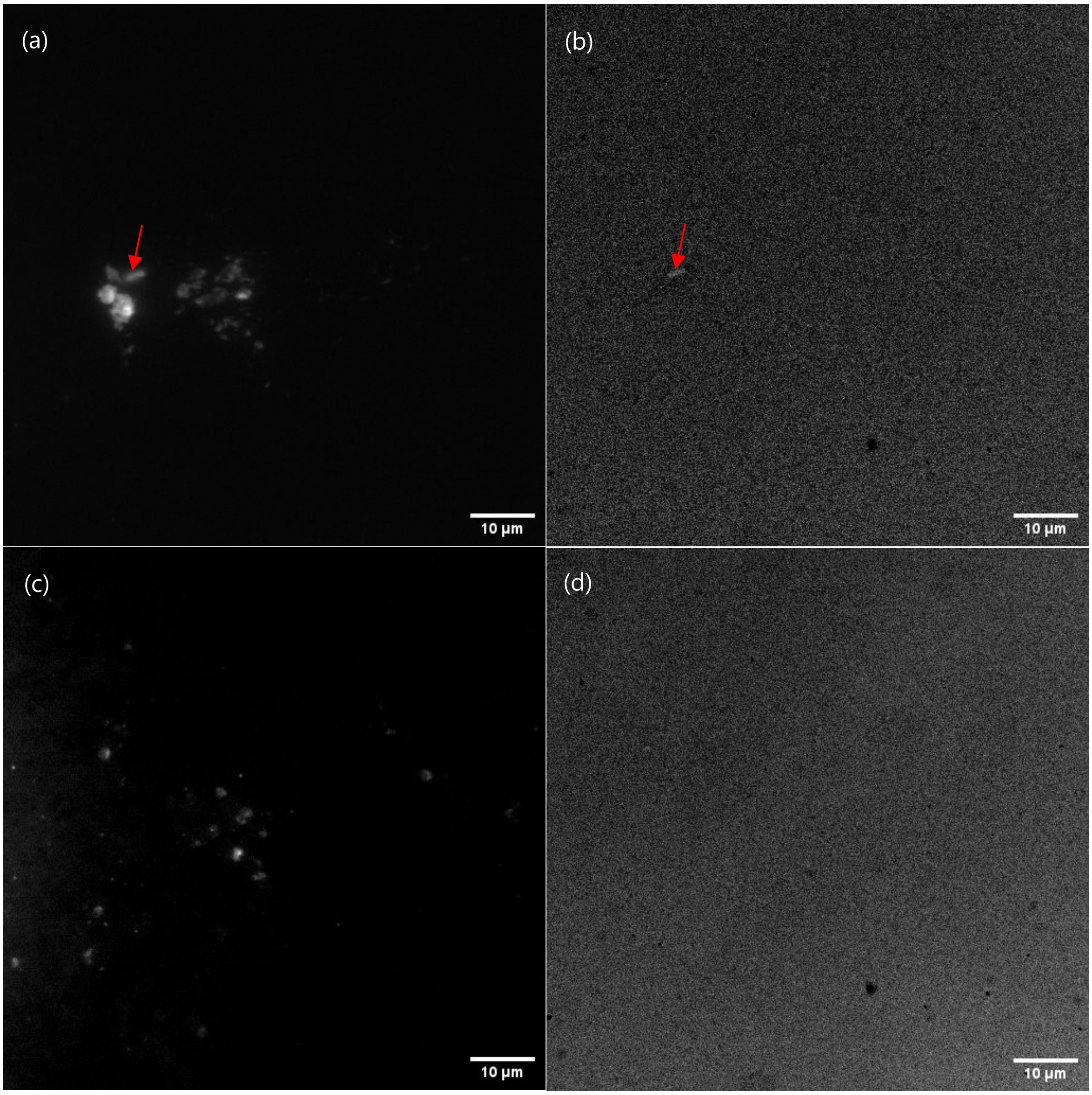

Figure 6: TIRF images (left and Brightfield images (right) of TO-CNF aerogels in the HEWL (a, b) and TCEP (c, d) solutions. Red arrows indicate fibre-like structures

7. SEM images

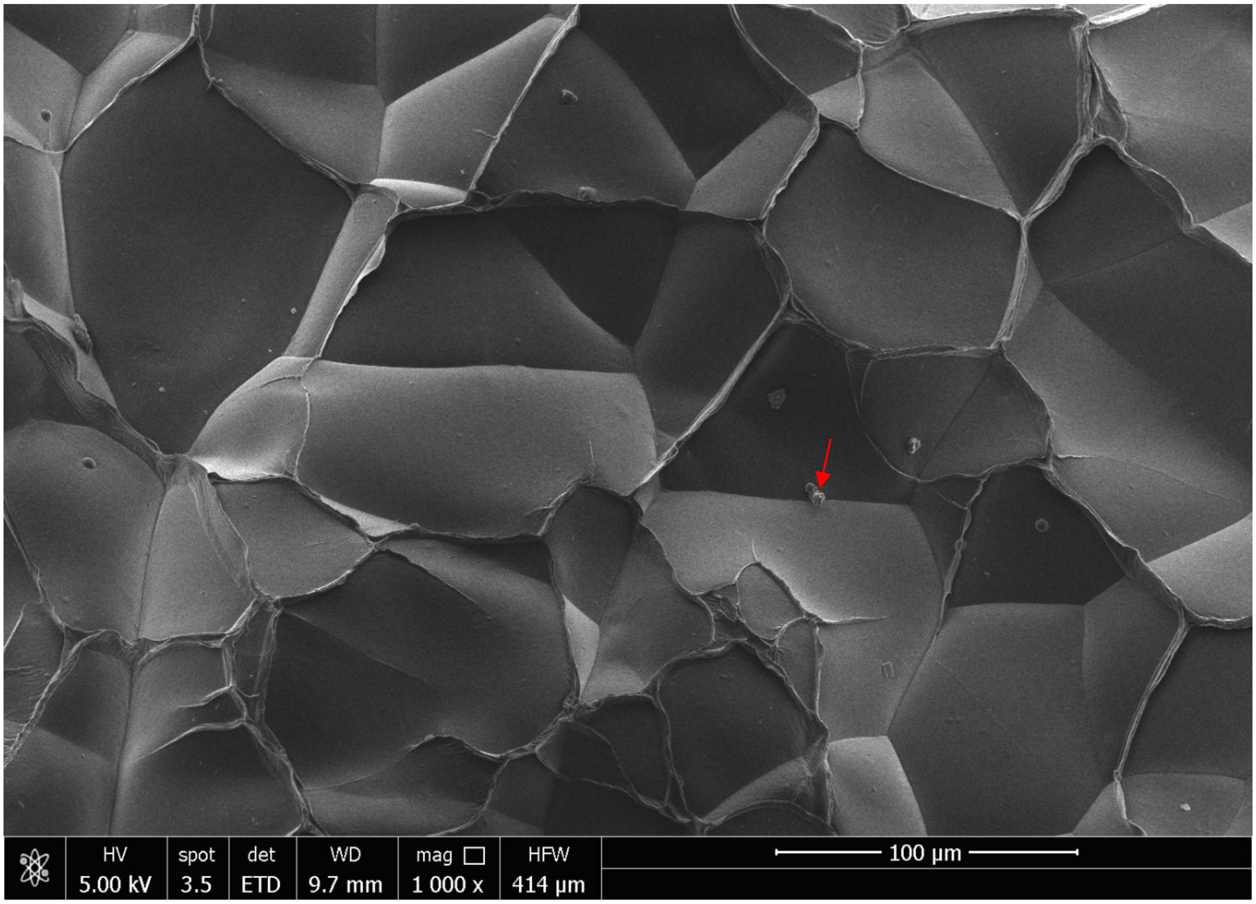

Figure 7: SEM image if TO-CNF aerogel incubated in a HEWL solution. Red arrow indicates protein agglomerates
